# Declining incidence rate of tuberculosis among close contacts in five years post-exposure: a systematic review and meta-analysis

**DOI:** 10.1186/s12879-023-08348-z

**Published:** 2023-06-03

**Authors:** Ying Du, Yijun He, Haoran Zhang, Fei Shen, Ling Guan, Henan Xin, Yongpeng He, Xuefang Cao, Boxuan Feng, Zhusheng Quan, Jianmin Liu, Lei Gao

**Affiliations:** 1 Beijing Key Laboratory of Clinical Epidemiology, School of Public Health, Capital Medical University, Beijing, 100069 China; 2 NHC Key Laboratory of Systems Biology of Pathogens, Institute of Pathogen Biology, and Center for Tuberculosis Research, Chinese Academy of Medical Sciences and Peking Union Medical College, No 9 Dong Dan San Tiao, Beijing, 100730 China; 3 The Sixth People’s Hospital of Zhengzhou, Zhengzhou, 400060 China

**Keywords:** Close contact, Incidence, Tuberculosis, Post-Exposure, Meta-analysis

## Abstract

**Background:**

Individuals in close contact with active pulmonary tuberculosis (TB) patients showed a high risk of recent infection and, once infected, higher risk of developing active TB in the following years post-exposure. But the peak time of active disease onset is unclear. This study aims to estimate post exposure TB incidence risk among close contacts to provide reference for clinical and public health strategies.

**Methods:**

We searched PubMed, Web of Science, and EMBASE for articles published until December 1, 2022. The incidence rates were quantitatively summarized by means of meta-analysis using the random-effect model.

**Results:**

Of the 5616 studies, 31 studies included in our analysis. For baseline close contacts results, the summarized prevalence of *Mycobacterium tuberculosis* (MTB) infection and active TB was found to be 46.30% (95% CI: 37.18%-55.41%) and 2.68% (95% CI: 2.02%-3.35%), respectively. During the follow-up, the 1-year, 2-year and 5-year cumulative incidence of TB in close contacts were 2.15% (95% CI: 1.51%-2.80%), 1.21% (95% CI: 0.93%-1.49%) and 1.11% (95% CI: 0.64%-1.58%), respectively. Individuals with a positive result of MTB infection testing at baseline showed significantly higher cumulative TB incidence as compared to those negatives (3.80% vs. 0.82%, *p* < 0.001).

**Conclusions:**

Individuals with close contact to active pulmonary TB patients are bearing significant risk of developing active TB, particularly within the first-year post-exposure. Population with recent infections should be an important priority for active case finding and preventive intervention worldwide.

**Supplementary Information:**

The online version contains supplementary material available at 10.1186/s12879-023-08348-z.

## Introduction

Globally, tens of millions of people might be exposed to *Mycobacterium tuberculosis* (MTB) each year [1–3]. According to the latest estimates by the World Health Organization (WHO),10.6 million people fell ill with TB worldwide, and 1.6 million people died from TB in 2021 [3]. Individuals in close contact with active pulmonary tuberculosis (TB) patients showed a high risk of recent infection and, once infected, a higher risk of developing active TB in the following two years post-exposure [1, 4, 5]. In many areas with a low incidence of TB, identifying and evaluating people who have come into contact with active TB patients was one important component of TB control programs [2]. WHO recommended MTB infection testing and treatment along with contact investigation for contacts of bacteriologically confirmed TB cases as well [3].

One of the purposes of conducting contact investigation is to find active cases earlier among close contacts of pulmonary TB disease, and another one is to identify the recently infected persons for further preventive treatment to protect them from developing active disease. One systematic review and meta-analysis included 41 studies reported that contact investigation was an effective tool of case finding [6]. Another meta-analysis which included 34 studies reported a summarized prevalence of active TB among household contacts was 2.3% (95% Confidence interval [CI]: 2.1%-2.5%) [7]. However, prospective studies addressing the incidence of active TB among close contacts in different periods of post-exposure were much less or long time ago [4, 5]. Therefore, the significance of expanding MTB infection testing and treatment among close contacts is not very clear in different settings with varied TB epidemics. In addition, identifying the peak time of active disease onset is also important for targeting at risk individuals for intervention more precisely. Therefore, this study aims to estimate the incidence of active TB among close contacts in different periods of post-exposure by means of systematic review and meta-analysis.

## Methods

The study protocol was prospectively registered in PROSPERO (number CRD42021265151). This systematic review and meta-analysis was conducted and reported in accordance with the Preferred Reporting Items for Systematic Reviews and Meta-Analyses (PRISMA) reporting standards [8].

### Information sources and search strategy

A systematic search of PubMed, Embase, and Web of Science was conducted from inception to December 1, 2022. Various combinations of the terms “tuberculosis”, “*mycobacterium tuberculosis*”, “contact tracing” and “close contact” were used to screen for potential studies reported TB incidence among contacts of active TB patients without any geographical restriction. The detailed search strategy was presented in Supplementary Files. Additional studies were also identified by cross-referencing.

### Eligibility criteria

Inclusion criteria include: the study should be a prospective or retrospective cohort study and studied contacts should be followed for at least one year; the study should report original data of TB incidence among close contacts during the follow-up period. Exclusion criteria include: non-English reports if the necessary information was not reported in the abstract in English; non-original articles with repeated or incomplete data; the sample size of the studied close contacts was less than 100 [9].

### Selection process

Two investigators Y Du and YJ He independently completed study screening. After duplications were removed, the two authors screened the studies in two stages: first by title and abstract and then by full text article. They independently finished study identification and data extraction, and consensus was reached on all of the items. Discrepancies were resolved by consensus with a third researcher (HR Zhang). All full texts against eligibility criteria were also checked by HR Zhang.

### Data extraction

Literature management used Endnote X9.3.3. When data were reported from overlapping study samples, the most recent and comprehensive reports were considered. The extracted data include: study information (first author, publication year, study design, demographics, number of index cases, number of close contact cases, age and gender distribution, diagnosis of index case, and exposure classification of contacts), baseline investigation information (diagnosis and prevalence of TB, diagnosis and prevalence of MTB infection), and follow-up investigation information (follow-up period, diagnosis and incidence of TB).

### Study definitions

Close contacts were defined as either household contacts or non-household contacts. Most of the included studies defined household contact as a person who had shared the same enclosed living space with the index case for more than one or more nights or frequent or extended daytime periods during the 3 months before the start of current treatment; most studies defined non-household as a person who was not in the household but shared an enclosed space (such as a social gathering, workplace or facility) for extended periods during the day with index case during the 3 months before the commencement of the current TB treatment episode. Most studies reported the incident TB during follow-up as confirmed cases without coprevalent disease, which was defined as confirmed TB identified at baseline or within 3 months post-exposure. Most studies took coprevalent disease into account to estimate the prevalence of active TB among close contacts at baseline. MTB infection status was defined as interferon-gamma release assay (IGRA) positive or a tuberculin skin test (TST) induration response of ≥ 10 mm in adults or ≥ 5 mm in children in most included studies. High TB burden countries were defined by WHO global TB report 2020 [3]. Only people who did not receive preventive chemotherapy were included in this analysis.

### Statistical analysis

Inter-study heterogeneity was assessed by Chi-square test with significance set at the *p* < 0.10 level. Higgins’ I2 statistics, values range from 0 to 100%, and values ≥ 50% were considered to be indicative of substantial heterogeneity [10]. Effect size with its 95% CI was calculated with a random-effects model when heterogeneity was considerable among studies. The age of index cases was presented with mean ± standard deviation (SD) or median (Q25-Q75). Stratified analyses were conducted according to the study origin (high TB burden countries or other countries), time-period of follow-up (1-year, 2-year and 5-year), MTB infection status at baseline (positive or negative), microbiologically status of the index case (positive). Egger weighted regression method, Begg rank correlation method [11] and symmetry of the funnel plots were used to assess the possibility of publication bias with significance at the *p* < 0.05 [12]. All the statistical analyses were performed through STATA 12.0 (Stata Corporation, College Station, TX, USA).

## Result

### Study identification and characteristics of the included studies

**Fig. 1:**
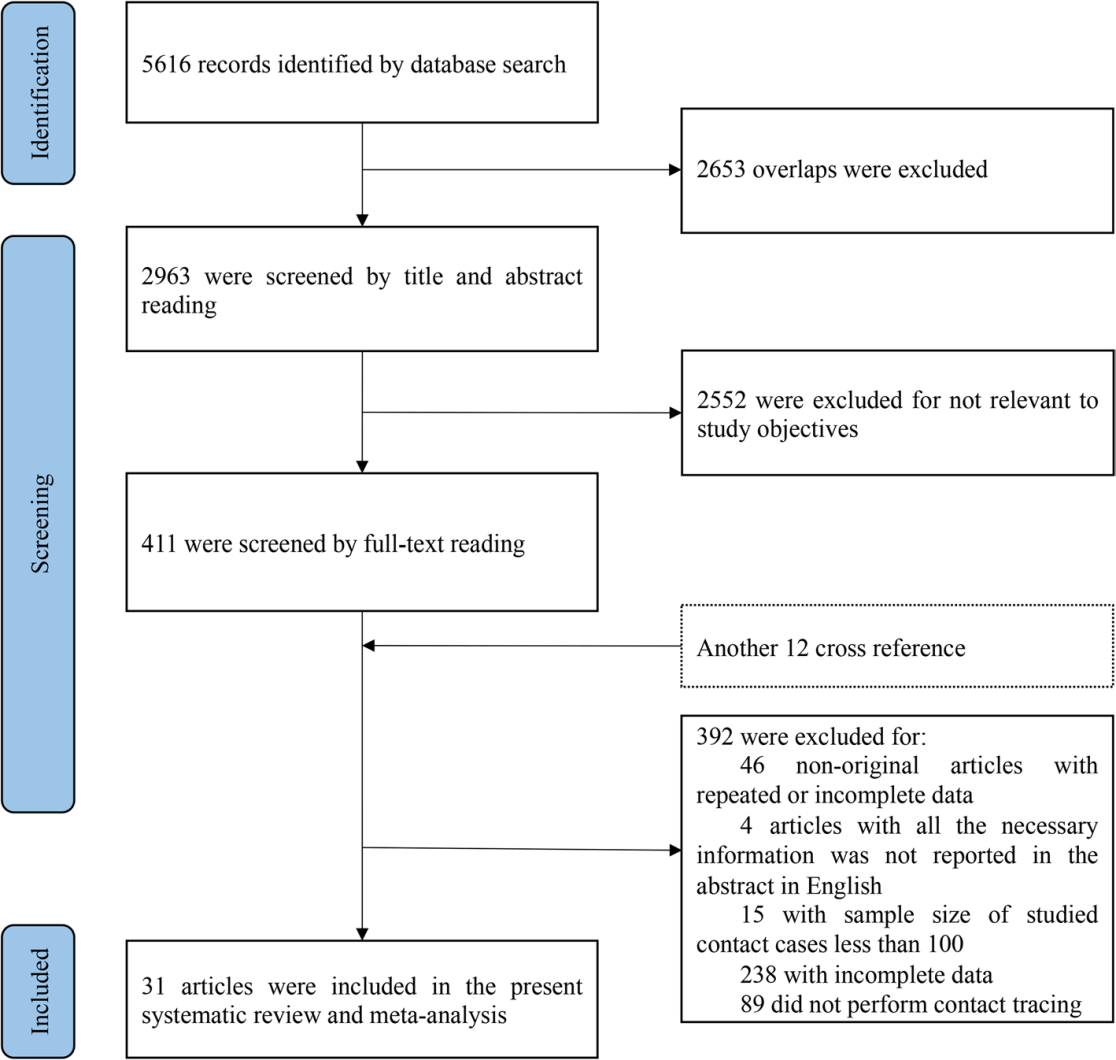
Identification of eligible studies on the incidence of active tuberculosis among close contacts. A total of 5,616 articles were obtained by database searches using different combinations of key terms. Among them, 2,653 records excluded because of overlap and 2,552 were excluded by abstracts review due to irrelevant to the study objective. Thus, 411 articles and another 12 cross-references were full text retrieved for more detailed evaluation. Of them, 46 non-original articles and 4 non-English articles were firstly excluded, and then 15 were excluded because the sample size of the studied contacts was less than 100, 238 were excluded because of missing completed incidence-related data, 89 were excluded because did not complete contact tracing, finally, a total of 31 eligible articles were included in this study

A total of 5,616 articles were obtained by database searches using different combinations of key terms (Fig. 1). Among them, 2,653 were excluded due to overlapping and 2,552 were excluded by abstract review due to irrelevant to the study objective. Thus, 411 articles and another 12 cross-references were full text retrieved for more detailed evaluation. Of them, 46 non-original articles (i.e. abstract, letter, editorial, poster, and review) and 4 non-English articles were firstly excluded, and then 15 were excluded because the sample size of the studied contact cases was less than 100, 238 were excluded because of missing completed incidence-related data, 89 were excluded because did not perform contact tracing, finally, a total of 31 eligible articles were included in this study [4, 5, 13–41] (Detailed search strategy and included studies were in Supplementary Appendix).

As shown in Table 1, publication of the included studies occurred between 1960 and 2020, and the sample size of contacts varied from 103 to 10,160. Of the 31 included studies, 21 (68%) were based on prospective design and 12 (39%) came from high TB burden countries. A total of 12,889 index cases and 53,327 contact cases were included in this analysis. For one bacteriologically confirmed pulmonary TB patient, the mean number of contacts was identified to be about 4. Most of the studied index cases were adults while their contact cases included both children and adults. Table 2 shows the included studies’ baseline results and follow-up investigation information among close contacts.

**Table 1:** Basic information of the included studies

First author, publish year	Study design	Study site	Index cases			Close contacts		
			No	Age (years) range/mean ± SD/ median(IQR), gender (%)	Diagnosis	No	Age (years) range/mean ± SD/ median(IQR), gender(%)	Exposure classification^a^
Yassin MA, 2020 [4]	Prospective	Ethiopia	344	35 (26–45), male (57)	Smear +	1,517	18 (12–30), male (57)	HHC
Araujo NCN, 2020 [13]	Retrospective	Brazil	1,672	NA	Microbiologically confirmed	1,264	9 (4–12), male (54)	HHC and non-HHC
Saunders MJ, 2019 [5]	Retrospective	Callao, Peru	715	27 (20–36), male (59)	Microbiologically confirmed	2,666	29 (21–42), male (46)	HHC
Huerga H, 2019 [14]	Prospective	Yerevan, Armenia	79	> 15, NA	Microbiologically confirmed	150	< 15, male (47)	HHC and non-HHC
Benjumea-Bedoya D, 2019 [15]	Prospective	Colombia	380	NA	Smear +	1,040	< 15, male (51)	HHC
Becerra MC, 2019 [16]	Prospective	Lima, Peru	2,516	> 16, NA	Microbiologically confirmed	10,160	All ages, male (45)	HHC
Reichler MR, 2018 [17]	Prospective	USA & Canada	718	> 15, NA	Microbiologically confirmed	4,490	NA	HHC and non-HHC
Martinez L, 2018 [18]	Prospective	Kampala, Uganda	857	> 18, NA	Microbiologically confirmed	1,718	< 16, male (33)	HHC and non-HHC
Baliashvili D, 2018 [19]	Retrospective	Gerogia, USA	896	41 ± 17, male (75)	Smear +	3,313	32 ± 21 male (43)	HHC and non-HHC
Sharma SK, 2017 [20]	Prospective	New Delhi, India	342	18–65, NA	Smear +	1,511	24 ± 15, male (52)	HHC
Puma DV, 2017 [21]	Retrospective	Catalonia, Spain	26	35 ± 17, male (66)	NA	103	29 ± 18, male (60)	HHC
Munoz L, 2017 [22]	Retrospective	Barcelona, Spain	360	NA	Microbiologically confirmed	661	37 (26–49), male (47)	HHC and non-HHC
Triasih R, 2015 [23]	Prospective	Indonesia	141	NA	Microbiologically confirmed	269	< 15, NA	HHC and non-HHC
Chakhaia T, 2014 [24]	Retrospective	Tbilisi, Georgia	396	≥ 5, male (63)	Microbiologically confirmed	869	All ages, male (43)	HHC and non-HHC
Singh J, 2013 [25]	Prospective	India	432	34 ± 14, male (58)	Microbiologically confirmed	1,608	27 ± 16, male (54)	HHC and non-HHC
Haldar P, 2013 [26]	Prospective	UK	505	NA	Microbiologically confirmed	1,671	≥ 16, male (50)	HHC and non-HHC
Wang JY, 2012 [27]	Prospective	China	NA	NA	NA	600	NA	HHC
Song S, 2012 [28]	Retrospective	South Korea	44	NA	Clinical confirmed / Microbiologically confirmed	1,826	18, male (51)	Non-HHC
Denholm JT, 2012 [29]	Retrospective	Victoria, Australia	47	NA	NA	570	28 (18–37), male (44)	Non-HHC
Becerra MC, 2011 [30]	Retrospective	Peru	693	NA	Microbiologically confirmed	4,503	25 ± 19, male (49)	HHC
Lienhardt C, 2010 [31]	Prospective	Dakar, Senegal	206	28 (18–71), male (68)	Smear +	2,762	20 (10–31), male (54)	HHC
del Corral H, 2009 [32]	Prospective	Colombia	366	36 (24–50), male (57)	Smear +	2,060	22 (10–42), male (43)	HHC
Cailleaus M, 2009 [33]	Retrospective	Brazil	276	NA	Microbiologically confirmed	1,178	≥ 12, male (38)	HHC and non-HHC
Hill PC, 2008 [34]	Prospective	Gambia	317	NA	Smear +	2,381	NA	HHC
Diel R, 2008 [35]	Prospective	Germany	107	28 ± 12, male (49)	Smear +	629	26 ± 14, NA	Non-HHC
Lemos AC, 2004 [36]	Prospective	Bahia, Brazil	69	29 ± 12, male (70)	Smear +	269	26 ± 17, male (50)	HHC
Bayona J, 2003 [37]	Prospective	Lima, Peru	192	NA, male (55)	Microbiologically confirmed	945	All ages, male (45)	HHC
Devadatta S, 1970 [38]	Prospective	Madras, India	NA	NA	NA	875	All ages, NA	HHC and non-HHC
Kamat SR, 1966 [39]	Prospective	Madras, India	NA	NA	NA	435	All ages, male (50)	HHC and non-HHC
Ramakrishnan CV, 1961 [40]	Prospective	Madras, India	NA	NA	NA	612	All ages, male (50)	HHC and non-HHC
Andrews RH, 1960 [41]	Prospective	Madras, India	193	NA	NA	672	All ages, male (50)	HHC and non-HHC

*Abbreviation*: *HHC* Household contact, *IQR* Inter quartile range, *NA* Not available, *SD* Standard deviation, *smear* + smear positive

^a^Close contact was defined as either household contacts or non-household contacts: most articles defined household contact as a person who had shared the same enclosed living space with the index case for more than one or more nights or for frequent or extended daytime periods during the 3 months before the start of current treatment; most studies defined non-household contact as a person who was not in the household but shared an enclosed space (such as a social gathering, workplace or facility) for extended periods during the day with index case during the 3 months before commencement of the current TB treatment episode

**Table 2:** Baseline and follow-up characteristics of close contacts of all included studies

First author, publish year	Baseline close contact survey				Incidence of active TB during follow up			
	TB prevalence^a^		MTB infection prevalence		Follow up period	Incidence of active TB during follow up(%)	TB Cases^b^	Close contacts (person years)
	n/N (%)	Diagnosis	n/N (%)	Testing				
Yassin MA, 2020 [4]	NA	Microbiologically confirmed / Clinically confirmed	NA	NA	1 year	2.74	41	1,496
					2 years	1.79	53	2,965
Araujo CN, 2020 [13]	20/495 (4.04)	Microbiologically confirmed/ Clinically confirmed	351/435 (80.69)	TST ≥ 5 mm	2 years	1.11	3	270
Saunders MJ, 2019 [5]	NA	Microbiologically confirmed/ Clinically confirmed	NA	NA	1 year	2.91	76	2,611
					2 years	2.36	121	5,130
					5 years	1.43	174	12,150
Huerga H, 2019 [14]	3/150 (2.00)	Microbiologically confirmed	NA	NA	2 years	0	0	246
Benjumea-Bedoya D, 2019 [15]	NA	Microbiologically confirmed/ Clinically confirmed	243/458 (53.06)	TST > 5 mm	2 years	1.49	31	2,080
Becerra MC, 2019 [16]	NA	Microbiologically confirmed/ Clinically confirmed	4,488/1,016 (44.17)	TST ≥ 10 mm	1 year	3.13	318	10,160
Reichler MR, 2018 [17]	108/4,490 (2.41)	Microbiologically confirmed	NA	TST ≥ 5 mm	1 year	1.45	64	4,419
					2 years	0.79	69	8,766
					5 years	0.35	75	21,654
Martinez L, 2018 [18]	126/1,718 (7.33)	Microbiologically confirmed/ Clinically confirmed	1,261/1,718 (70.78)	TST ≥ 5 mm	2 years	0.70	24	3,436
Baliashvili D, 2018 [19]	64/3,133 (2.04)	Microbiologically confirmed	393/1,157 (33.97)	TST ≥ 10 mm	1 year	1.66	52	3,133
Sharma SK, 2017 [20]	NA	Microbiologically confirmed/ Clinically confirmed	787/1,511 (52.08); 917/1,511 (20.09)	TST ≥ 10 mm /IGRA +	2 years	2.51	76	3,022
Puma DV, 2017 [21]	NA	NA	38/103 (36.89)	TST ≥ 5 mm	5 years	0.19	1	515
Munoz L, 2017 [22]	NA	NA	223/321(69.47); 184/340(54.11)	TST ≥ 10 mm /IGRA +	5 years	0.09	3	3305
Triasih R, 2015 [23]	25/269 (9.29)	Microbiologically confirmed	100/269 (37.17)	TST ≥ 10 mm	1 year	2.76	4	145
Chakhaia T, 2014 [24]	30/869 (3.45)	Microbiologically confirmed	212/402 (52.74)	TST ≥ 5 mm	1 years	1.08	9	833
					2 years	1.32	17	1,285
Singh J, 2013 [25]	52/1,608 (3.23)	Microbiologically confirmed	NA	NA	1 years	1.41	27	1,914
					2 years	0.86	31	3,621
Haldar P, 2013 [26]	6/1,671 (0.36)	Microbiologically confirmed/ Clinically confirmed	215/851 (25.26)	IGRA +	2 years	1.29	43	3,342
Wang JY, 2012 [27]	17/600 (2.83)	NA	176/583 (30.19)	IGRA +	2 years	0.82	9	1,102
Song S, 2012 [28]	NA	Microbiologically confirmed/ Clinically confirmed	270/1,826 (14.79)	TST ≥ 10 mm	2 years	0.48	16	3,310
Denholm JT, 2012 [29]	NA	NA	49/552 (8.86)	TST ≥ 10 mm	2 years	0.18	2	1,118
Becerra MC, 2011 [30]	117/4,503 (2.60)	NA	NA	NA	1 year	3.17	140	4,423
					2 years	2.14	187	8,726
Lienhardt C, 2010 [31]	14/2,762 (0.51)	Microbiologically confirmed/ Clinically confirmed	1,591/2,458 (64.73)	TST ≥ 10 mm	2 years	0.93	52	5,606
del Corral H, 2009 [32]	8/2,060 (0.39)	NA	331/502 (65.94); 1,310/1,977 (66.26)	TST ≥ 10 mm/ IGRA +	2 years	0.94	37	3,934
Cailleaus M, 2009 [33]	NA	Clinically confirmed	460/998 (46.09)	TST ≥ 5 mm	2 years	1.65	22	1,334
Hill PC, 2008 [34]	33/2,381 (1.39)	Microbiologically confirmed/ Clinically confirmed	NA	NA	1 years	0.64	15	2,326
					2 years	0.58	26	4,518
Diel R, 2008 [35]	3/629 (0.48)	Microbiologically confirmed	110/601 (18.30); 66/601 (10.98)	TST ≥ 10 mm/ IGRA +	2 years	0.43	5	1,150
Lemos AC, 2004 [36]	7/269 (2.6)	NA	136/269 (50.56)	TST ≥ 10 mm	1 year	1.12	3	269
Bayona J, 2003 [37]	29/945 (3.07)	NA	NA	NA	5 years	1.57	72	4,590
Devadatta S, 1970 [38]	NA	Microbiologically confirmed/ Clinically confirmed	NA	NA	5 years	1.76	77	4,375
Kamat SR, 1966 [39]	NA	Microbiologically confirmed/ Clinically confirmed	NA	NA	5 years	2.85	62	2,175
Ramakrishnan CV, 1961 [40]	NA	Microbiologically confirmed/ Clinically confirmed	NA	NA	2 years	2.78	34	1,224
Andrews RH, 1960 [41]	50/693 (7.22)	Microbiologically confirmed	455/647 (70.32)	TST ≥ 5 mm	1 year	5.01	32	639

*Abbreviation*: *IGRA* Interferon gamma release assay, *IGRA* + IGRA positive, *MTB Mycobacterium tuberculosis*, *NA* Not available, *TB* Tuberculosis, *TST* Tuberculin skin test

^a^Most articles reported active TB prevalence at baseline among close contacts including coprevalent disease which was defined as confirmed TB identified at baseline or within 3 months post exposure

^b^Most articles defined incidence of active TB during follow up as incident TB without coprevalent disease

### Meta-analysis of active TB incidence among close contacts

Table 3 shows the summarized active TB incidence among close contacts during the follow-up. The 1-year, 2-year and 5-year cumulative incidence rate of active TB in close contacts not receiving preventive therapy was found to be 2.15% (95% CI: 1.51%-2.80%), 1.21% (95% CI: 0.93%-1.49%) and 1.11% (95% CI: 0.64%-1.58%), respectively. Supplementary Fig. 1 showed there was no significant publication bias for 1-year cumulative incidence (Egger’s test, *p* = 0.448 and Begg’s test, *p* = 0.086), but the funnel plot seemed to be asymmetric (Supplementary Fig. 2). The 2-year active TB incidence of close contacts who exposed to TB patients with sputum smear-positives was 1.55% (95% CI: 1.08%-2.02%). Close contacts with a positive result of MTB infection testing at baseline post-exposure showed significantly higher 2-year cumulative TB incidence as compared to those negatives (3.80% vs. 0.82%, *p* < 0.001). Close contacts from high TB-burden countries showed higher 2-year cumulative TB incidence than those from other countries (1.63% vs. 1.03%), but the difference was not statistically significant (*p* = 0.057).

**Table 3:** Meta-analysis of active tuberculosis incidence among close contacts during follow-up

Subgroups	Follow-up period	No. of included studies^a^	Summarized incidence^b^ (95% CI)	Heterogeneity	
				I^2^	*P* for Q test
Total	1 year	12	**2.15% (1.51%-2.80%)**	93.72%	< *0.0001*
	2 years	21	1.21% (0.93%-1.49%)	92.40%	< *0.0001*
	5 years	7	1.11% (0.64%-1.58%)	97.73%	< *0.0001*
Index cases were smear +	2 years	6	1.55% (1.08%-2.02%)	87.13%	< *0.0001*
Baseline MTB infection testing + ^c^	2 years	12	3.80% (2.74%-4.87%)	81.86%	< *0.0001*
Baseline MTB infection testing–	2 years	9	0.82% (0.47%-1.18%)	79.65%	< *0.0001*
From high TB burden countries^d^	2 years	7	1.63% (1.04%-2.21%)	86.46%	< *0.0001*
From other countries^d^	2 years	14	1.03% (0.71%-1.35%)	93.46%	< *0.0001*

*Abbreviation*: *CI* Confidence interval, *MTB Mycobacterium tuberculosis*

^a^Only people who did not receive preventive chemotherapy were included in this analysis

^b^Most articles reported the incident tuberculosis (TB) as confirmed cases without coprevalent disease which was defined as confirmed or unconfirmed TB identified at baseline or within 3 months post-exposure

^c^Most studies’ baseline MTB infection testing + was defined as a tuberculin skin-test (TST) induration response of ≥ 10 mm in adults or ≥ 5 mm in children. One study [26] defined as QuantiFERON Gold In-Tube Test and one [27] defined as T-SPOT.TB

^d^High TB burden countries were defined by World Health Organization global TB report 2022 (Geneva: World Health Organization, 2022) [3]

### Meta-analysis of active TB prevalence and MTB infection prevalence among close contacts post-exposure

Of the 31 included cohort studies, 18 studies reported the baseline TB prevalence among close contacts post-exposure and the summarized estimation was 2.68% (95% CI: 2.02%-3.35%). Studies participants from high TB burden countries showed a higher baseline prevalence of TB than those from other countries (5.55% vs. 2.16%, *p* = 0.022) (Table 4).

**Table 4:** Meta-analysis of active tuberculosis prevalence and *Mycobacterium tuberculosis* infection prevalence at baseline among close contacts

Subgroups	No. of included studies^a^	Summarized prevalence of active TB^b^ (95% CI)	Heterogeneity	
			I^2^	*P* for Q test
Total	18	2.68% (2.02%-3.35%)	95.67%	< *0.0001*
From high TB burden countries^e^	4	5.55% (2.98%-8.11%)	85.90%	< *0.0001*
From other countries^e^	14	2.16% (1.49%-2.82%)	95.89%	< *0.0001*
Subgroups	No. of included studies^c^	Summarized prevalence of MTB infection (95% CI)	Heterogeneity	
			I^2^	*P* for Q test
Total	20	46.30% (37.18%-55.41%)^d^	99.58%	< *0.0001*
Testing by TST	18	48.37% (38.58%-58.16%)	99.61%	< *0.0001*
Testing by IGRA	6	41.24% (21.06%-61.42%)	99.67%	< *0.0001*
Index cases were smear +	5	53.94% (41.76%-66.12%)	98.90%	< *0.0001*
From high TB burden countries^e^	6	52.80% (38.62%-66.98%)	98.99%	< *0.0001*
From other countries^e^	14	43.51% (32.32%-54.71%)	99.65%	< *0.0001*

*Abbreviation CI* Confidence interval, *IGRA* Interferon gamma release assay, *MTB* Mycobacterium tuberculosis, *TB* Tuberculosis, *TST* Tuberculin skin test

^a^Of the included 31 studies, 18 of them investigating the baseline active tuberculosis (TB) prevalence were included in summarized active TB prevalence analysis

^b^Most articles’ active TB prevalence at baseline among close contacts included coprevalent disease which was defined as confirmed or unconfirmed TB identified at baseline or within 3 months post-exposure

^c^Of the included 31 studies, 20 of them investigating the baseline Mycobacterium tuberculosis infection (MTB) infection prevalence were included in Summarized MTB infection prevalence analysis. Most studies’ MTB infection was defined as a tuberculin skin-test induration response of ≥ 10 mm in adults or ≥ 5 mm in children. Several articles defined as both TST and IGRA

^d^MTB infection was defined as TST ≥ 10 mm in adults or ≥ 5 mm in children other than only IGRA were used [26, 27]

^e^High TB burden countries were defined by World Health Organization global TB report 2022 (Geneva: World Health Organization, 2022) [3]

In addition, 20 of the 31 included cohort studies reported the baseline MTB infection prevalence among close contacts post-exposure (Table 4), the summarized estimation was 46.30% (95% CI: 37.18%-55.41%). In 18 studies, TST was used to test MTB infection and the summarized prevalence of MTB infection was 48.37% (95% CI: 38.58%-58.16%). While 6 studies used IGRA to test MTB infection and the summarized prevalence was 41.24% (95% CI: 21.06%-61.42%). No statistically significant difference was observed between the summarized estimates based on these two tests (*p* = 0.571). In stratified analysis, the prevalence of MTB infection among close contacts of sputum smear-positive index cases was 53.94% (95% CI: 41.76%-66.12%). No statistically significant difference in summarized MTB infection prevalence among the close contacts was observed between studies from high TB burden countries and other countries included in the review(52.80% vs. 43.51%, *p* = 0.433).

## Discussion

The present study systematically reviewed the published cohort studies addressing the risk of active TB development among close contacts post-exposure. We found the cumulative incidence of active TB among close contacts was very high especially within the first-year post-exposure. The stratified analyses showed that contacts exposed to microbiologically confirmed pulmonary TB patients should be given priority for active TB screening and MTB infection testing and treatment, especially in the areas with high TB burden.

According to the WHO’s recommendations for middle- and low-income countries [42, 43], besides children younger than 5 years and people living with HIV, other risk populations such as close contacts of TB patients should also be screened to find active TB. It has been a national strategy in China to conduct active TB case tracing and provide free screening tests to close contacts of smear-positive TB patients since 2006 (Bureau of disease prevention and control under the ministry of health, 2006) [44]. This work has been systematically evaluated to some extent, but the national data was still scarce. Therefore, it is urgently needed to develop national guidelines providing precise intervention tools and standards for protecting close contacts from MTB infection and active disease development. After exposure, close contacts might present varied outcomes depending on their immune status and degree of exposure (exposure duration, disease severity of the index cases, etc.), including infection, infection clearance, and post-infection morbidity [45]. Therefore, under the influence of many factors, it is a challenge to carry out precise interventions based on an individual’s risk. Our meta-analysis results supported the findings of several individual reports that the first-year post-exposure was the peak period of developing the active disease for close contacts, which is similar to the findings of 2-year post-exposure was estimated as a high-risk period [4, 5, 17, 25, 30, 46]. The results of the meta-analysis are very helpful to define the screening priority more precisely, especially in developing or underdeveloped countries with limited resources.

We found few studies reported TB incidence among close contacts whose index case was smear negative. Haldar P [26] found that the 2-year TB incidence among close contacts was 2.6% for those exposed to sputum smear-positive index cases and only 0.7% for those exposed to sputum smear-negative index cases. On the contrary, Triasih R [23] and Guo J [47] found that TB incidence density among close contacts was not significantly different concerning the microbiological status of the index cases. In this review, there was no significant difference in active TB incidence across the baseline characteristics of close contact with microbiologically confirmed patients. Varied study populations and different TB epidemics in the study area might contribute to the heterogeneity observed between the different studies. However, based on the results of our review of MTB infection prevalence in post-exposure close contact populations, there are still reasons to believe that investigations among close contacts of people with bacteriologically confirmed pulmonary TB should be intensified. It should be a cost-effective option both for the early TB case finding and for implement of preventive treatment among close contacts.

TB is now understood as a dynamic multistate gradient from infection acquisition to subclinical disease and clinically active disease. The outcome of exposure to active TB patients was determined by a complex interaction of bacterial, host, and environmental factors [48]. For the host, the initial exposure gradient, such as bacterial load, disease severity of the index case, and the closeness and duration of the contact, were directly associated with the risk of developing primary disease [49]. In addition, the endogenous recurrence of TB was found to be mainly associated with weakened immunity of the host [50]. Individuals with a positive result of MTB infection testing post-exposure were found under significantly higher risk of active TB incidence as compared to those negatives. Similarly, in one meta-analysis included 46 cohort studies on exposed children [9], children with a positive result for MTB infection had significantly higher 2-year cumulative TB incidence than those negatives. MTB infection testing was essential for determining the priority individuals for MTB infection screening and preventive therapy in most populations except for immunocompromised ones such as HIV infections. Because the currently available MTB infection testing, TST and IGRA, were both immunological methods with limited sensitivity in the application of immune deficiency populations. Therefore, WHO recommended TB preventive treatment for HIV infections and children aged < 5 years who are household contacts even if MTB infection testing is unavailable [51]. However, MTB infection testing should be a priority action for the general population with close contact, it has been suggested to be a good practice to identify recent conversions before initiating TB preventive treatment [52].

## Limitations

There are several limitations in this review. First, the pooled analysis of all studies showed substantial heterogeneity across studies. Second, the literature describing the results of contact investigations were not easy to be systematically summarized. The quality of some literature was poor and the critical information for estimating incidence might frequently be missed. Such studies might be excluded which made our study results generalization limited. Also, some retrospective studies were included in this review, they could be attributed to, at least partially, to the presence of confounders, which then lower the power of generalization of the findings. Third, missing essential data also limited more detailed stratified analyses, such as many studies only reported 2-year TB incidence post-exposure, but 1-year estimate were less reported. These three databases cannot cover all related studies, and non-English reports were excluded if the necessary information was not reported in the abstract in English, such eligibility criteria might cause selection bias.

## Conclusion

Our review suggested that close contacts of patients with microbiologically confirmed pulmonary TB are a group at high risk of developing active TB, particularly within the first-year post-exposure. It may imply that expanding close contacts investigation in more at-risk populations is of great significance both for the early detection of TB and for precisely identifying MTB infection treatment targets. In addition, we should continuously optimize the guidelines to improve the quality of close contacts management combined with local public health resources and the TB epidemic situation.

## Supplementary Information

**Additional file 1. **

**Additional file 2. **

**Additional file 3. **

## Data Availability

All data generated or analyzed during this study are included in this published article and its supplementary information files.

## Abbreviations

TB: Tuberculosis
MTB: Mycobacterium tuberculosis
WHO: World Health Organization
CI: Confidence interval
IGRA: Interferon gamma release assay
TST: Tuberculin skin test
