# Supplementary material for: Declining incidence rate of tuberculosis among close contacts in five years post-exposure: a systematic review and meta-analysis
Source: BMC Infect Dis. 2023 Jun 3;23:373. doi: 10.1186/s12879-023-08348-z (PMC10239604; doi:10.1186/s12879-023-08348-z)
Supplement: Supplementary file 1 — Additional file 1. [file 12879_2023_8348_MOESM1_ESM.docx]

- **Search strategy**:

**The final PubMed search strategy:**

(((tuberculosis) OR (mycobacterium tuberculosis[MeSH Terms])) OR (tuberculosis[ MeSH Terms])) AND

((contact tracing[MeSH Terms]) OR (close contact)) Filters: Engelish

**The final Embase search strategy:**

#1 AND #2 AND English (language)

#1 (TS=(tuberculosis)) OR TS=(mycobacterium tuberculosis) OR TS=(TB)

#2 (TS=(‘contact tracing’)) OR TS=(close contact)

**The final Web of science search strategy:**

#4 AND #7 AND [english]/lim AND [medline]/lim

#4: #1 OR #2 OR #3

#7: #5 OR #6

#1 ‘tuberculosis’

#2 ‘tuberculosis’:de

#3 ‘mycobacterium tuberculosis’:de

#5 ‘contact examination’:de

#6 ‘close contact’

- **All incorporated references in supplementary:**

1. Yassin MA, Yirdaw KD, Datiko DG, Cuevas LE, Yassin MA. Yield of household contact investigation of patients with pulmonary tuberculosis in southern Ethiopia. BMC Public Health 2020; 20(1): 737. https://doi.org/ 10.1186/s12889-020-08879-z.
2. Araújo NCN, Cruz CMS, Arriaga MB, Cubillos-Angulo JM, Rocha MS, Silveira-Mattos PS, et al. Determinants of losses in the latent tuberculosis cascade of care in Brazil: A retrospective cohort study. Int J Infect Dis 2020; 93: 277-83. https://doi.org/ 10.1016/j.ijid.2020.02.015.
3. Saunders MJ, Tovar MA, Collier D, Baldwin MR, Montoya R, Valencia TR, et al. Active and passive case-finding in tuberculosis-affected households in Peru: a 10-year prospective cohort study. Lancet Infect Dis 2019; 19(5): 519-28. https://doi.org/ 10.1016/s1473-3099(18)30753-9.
4. Huerga H, Sanchez-Padilla E, Melikyan N, Atshemyan H, Hayrapetyan A, Ulumyan A, et al. High prevalence of infection and low incidence of disease in child contacts of patients with drug-resistant tuberculosis: a prospective cohort study. Arch Dis Child 2019; 104(7): 622-8. https://doi.org/ 10.1136/archdischild-2018-315411.
5. Benjumea-Bedoya D, Marín DM, Robledo J, Barrera LF, López L, Del Corral H, et al. Risk of infection and disease progression in children exposed to tuberculosis at home, Colombia. Colomb Med (Cali) 2019; 50(4): 261-74. https://doi.org/ 10.25100/cm.v50i4.4185.
6. Becerra MC, Huang CC, Lecca L, Bayona J, Contreras C, Calderon R, et al. Transmissibility and potential for disease progression of drug resistant Mycobacterium tuberculosis: prospective cohort study. BMJ (Clinical research ed) 2019; 367: l5894. https://doi.org/ 10.1136/bmj.l5894.
7. Reichler MR, Khan A, Sterling TR, Zhao H, Moran J, McAuley J, et al. Risk and Timing of Tuberculosis Among Close Contacts of Persons with Infectious Tuberculosis. J Infect Dis 2018; 218(6): 1000-8. https://doi.org/ 10.1093/infdis/jiy265.
8. Martinez L, Shen Y, Handel A, Chakraburty S, Stein CM, Malone LL, et al. Effectiveness of WHO's pragmatic screening algorithm for child contacts of tuberculosis cases in resource-constrained settings: a prospective cohort study in Uganda. Lancet Respir Med 2018; 6(4): 276-86. https://doi.org/ 10.1016/s2213-2600(17)30497-6.
9. Baliashvili D, Kempker RR, Blumberg HM, Kuchukhidze G, Merabishvili T, Aslanikashvili A, et al. A population-based tuberculosis contact investigation in the country of Georgia. Public Health Action 2018; 8(3): 110-7. https://doi.org/ 10.5588/pha.18.0024.
10. Sharma SK, Vashishtha R, Chauhan LS, Sreenivas V, Seth D. Comparison of TST and IGRA in Diagnosis of Latent Tuberculosis Infection in a High TB-Burden Setting. PLoS One 2017; 12(1): e0169539. https://doi.org/ 10.1371/journal.pone.0169539.
11. Puma DV, Pérez-Quílez O, Roure S, Martínez-Cuevas O, Bocanegra C, Feijoo-Cid M, et al. Risk of Active Tuberculosis among Index Case of Householders-A Long-Term Assessment after the Conventional Contacts Study. Public Health Nurs 2017; 34(2): 112-7. https://doi.org/ 10.1111/phn.12279.
12. Muñoz L, Gonzalez L, Soldevila L, Dorca J, Alcaide F, Santin M. QuantiFERON®-TB Gold In-Tube for contact screening in BCG-vaccinated adults: A longitudinal cohort study. PLoS One 2017; 12(8): e0183258. https://doi.org/ 10.1371/journal.pone.0183258.
13. Triasih R, Robertson C, Duke T, Graham SM. Risk of infection and disease with Mycobacterium tuberculosis among children identified through prospective community-based contact screening in Indonesia. Trop Med Int Health 2015; 20(6): 737-43. https://doi.org/ 10.1111/tmi.12484.
14. Chakhaia T, Magee MJ, Kempker RR, Gegia M, Goginashvili L, Nanava U, et al. High utility of contact investigation for latent and active tuberculosis case detection among the contacts: a retrospective cohort study in Tbilisi, Georgia, 2010-2011. PLoS One 2014; 9(11): e111773. https://doi.org/ 10.1371/journal.pone.0111773.
15. Singh J, Sankar MM, Kumar S, Gopinath K, Singh N, Mani K, et al. Incidence and prevalence of tuberculosis among household contacts of pulmonary tuberculosis patients in a peri-urban population of South Delhi, India. PLoS One 2013; 8(7): e69730. https://doi.org/ 10.1371/journal.pone.0069730.
16. Haldar P, Thuraisingam H, Patel H, Pereira N, Free RC, Entwisle J, et al. Single-step QuantiFERON screening of adult contacts: a prospective cohort study of tuberculosis risk. Thorax 2013; 68(3): 240-6. https://doi.org/ 10.1136/thoraxjnl-2011-200956.
17. Wang JY, Shu CC, Lee CH, Yu CJ, Lee LN, Yang PC. Interferon-gamma release assay and Rifampicin therapy for household contacts of tuberculosis. J Infect 2012; 64(3): 291-8. https://doi.org/ 10.1016/j.jinf.2011.11.028.
18. Song S, Jeon D, Kim JW, Kim YD, Kim SP, Cho JS, et al. Performance of confirmatory interferon-γ release assays in school TB outbreaks. Chest 2012; 141(4): 983-8. https://doi.org/ 10.1378/chest.11-1158.
19. Denholm JT, Leslie DE, Jenkin GA, Darby J, Johnson PD, Graham SM, et al. Long-term follow-up of contacts exposed to multidrug-resistant tuberculosis in Victoria, Australia, 1995-2010. Int J Tuberc Lung Dis 2012; 16(10): 1320-5. https://doi.org/ 10.5588/ijtld.12.0092.
20. Becerra MC, Appleton SC, Franke MF, Chalco K, Arteaga F, Bayona J, et al. Tuberculosis burden in households of patients with multidrug-resistant and extensively drug-resistant tuberculosis: a retrospective cohort study. Lancet 2011; 377(9760): 147-52. https://doi.org/ 10.1016/s0140-6736(10)61972-1.
21. Lienhardt C, Fielding K, Hane AA, Niang A, Ndao CT, Karam F, et al. Evaluation of the prognostic value of IFN-gamma release assay and tuberculin skin test in household contacts of infectious tuberculosis cases in Senegal. PLoS One 2010; 5(5): e10508. https://doi.org/ 10.1371/journal.pone.0010508.
22. del Corral H, París SC, Marín ND, Marín DM, López L, Henao HM, et al. IFNgamma response to Mycobacterium tuberculosis, risk of infection and disease in household contacts of tuberculosis patients in Colombia. PLoS One 2009; 4(12): e8257. https://doi.org/ 10.1371/journal.pone.0008257.
23. Cailleaux-Cezar M, de AMD, Xavier GM, de Salles CL, de Mello FC, Ruffino-Netto A, et al. Tuberculosis incidence among contacts of active pulmonary tuberculosis. Int J Tuberc Lung Dis 2009; 13(2): 190-5. https://doi.org/ 10.1371/journal.pone.0001379.
24. Hill PC, Jackson-Sillah DJ, Fox A, Brookes RH, de Jong BC, Lugos MD, et al. Incidence of tuberculosis and the predictive value of ELISPOT and Mantoux tests in Gambian case contacts. PLoS One 2008; 3(1): e1379. https://doi.org/ 10.1371/journal.pone.0001379.
25. Diel R, Loddenkemper R, Meywald-Walter K, Niemann S, Nienhaus A. Predictive value of a whole blood IFN-gamma assay for the development of active tuberculosis disease after recent infection with Mycobacterium tuberculosis. Am J Respir Crit Care Med 2008; 177(10): 1164-70. https://doi.org/ 10.1164/rccm.200711-1613OC.
26. Lemos AC, Matos ED, Pedral-Sampaio DB, Netto EM. Risk of tuberculosis among household contacts in Salvador, Bahia. Braz J Infect Dis 2004; 8(6): 424-30. https://doi.org/ 10.1590/s1413-86702004000600006.
27. Bayona J, Chavez-Pachas AM, Palacios E, Llaro K, Sapag R, Becerra MC. Contact investigations as a means of detection and timely treatment of persons with infectious multidrug-resistant tuberculosis. Int J Tuberc Lung Dis 2003; 7(12 Suppl 3): S501-9.
28. Devadatta S, Dawson JJ, Fox W, Janardhanam B, Radhakrishna S, Ramakrishnan CV, et al. Attack rate of tuberculosis in a 5-year period among close family contacts of tuberculous patients under domiciliary treatment with isoniazid plus PAS or isoniazid alone. Bull World Health Organ 1970; 42(3): 337-51.
29. Kamat SR, Dawson JJ, Devadatta S, Fox W, Janardhanam B, Radhakrishna S, et al. A controlled study of the influence of segregation of tuberculous patients for one year on the attack rate of tuberculosis in a 5-year period in close family contacts in South India. Bull World Health Organ 1966; 34(4): 517-32.
30. Ramakrishnan CV, Andrews RH, Devadatta S, Fox W, Radhakrishna S, Somasundaram PR, et al. Influence of segregation to tuberculous patients for one year on the attack rate of tuberculosis in a 2-year period in close family contacts in South India. Bull World Health Organ 1961; 24(2): 129-48.
31. Andrews RH, Devadatta S, Fox W, Radhakrishna S, Ramakrishnan CV, Velu S. Prevalence of tuberculosis among close family contacts of tuberculous patients in South India, and influence of segregation of the patient on early attack rate. Bull World Health Organ 1960; 23(4-5): 463-510.
