# Supplementary figures and images for: Declining incidence rate of tuberculosis among close contacts in five years post-exposure: a systematic review and meta-analysis
Source: BMC Infect Dis. 2023 Jun 3;23:373. doi: 10.1186/s12879-023-08348-z (PMC10239604; doi:10.1186/s12879-023-08348-z)

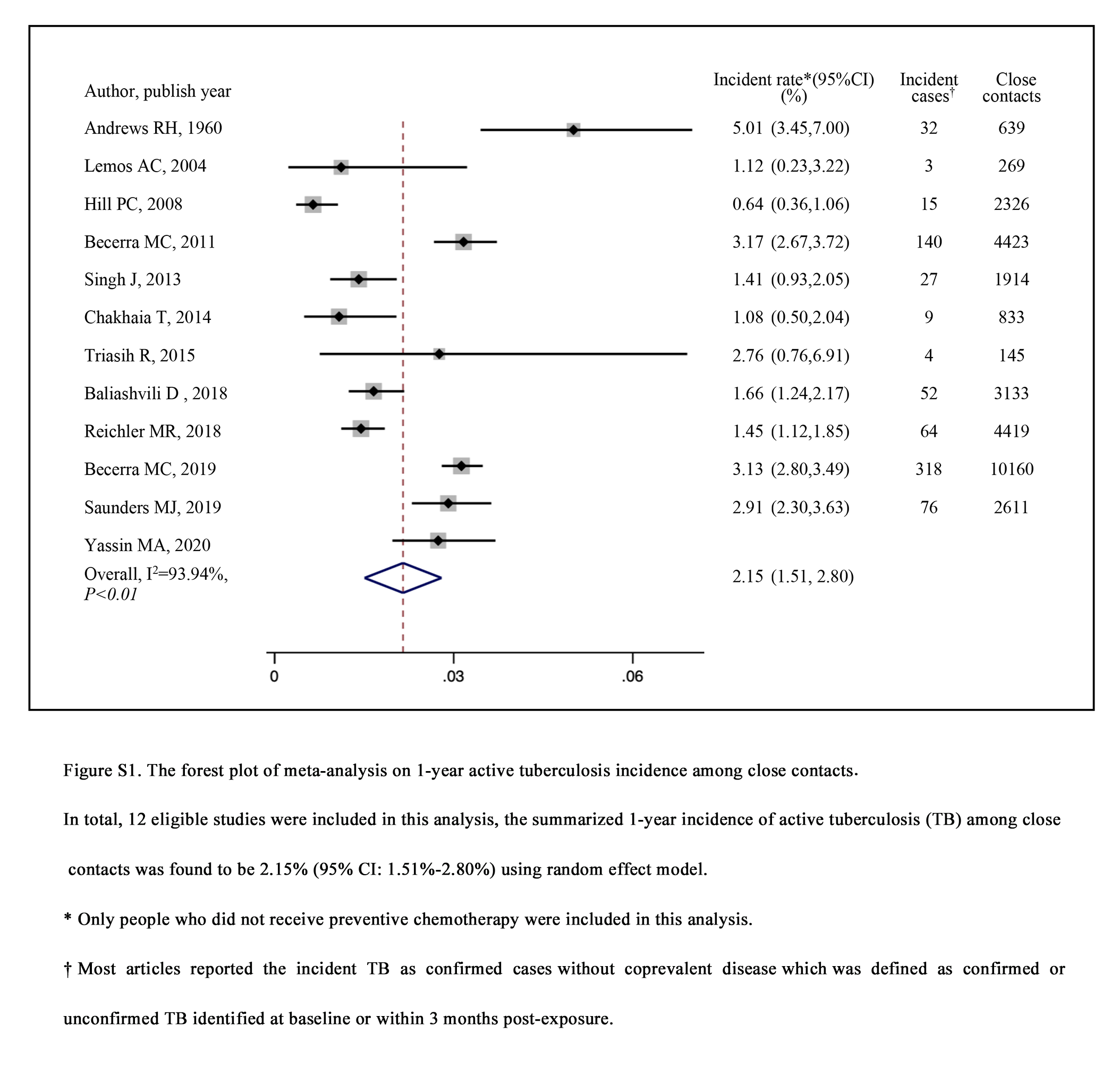

Supplement: Supplementary file 2 — Additional file 2. [file 12879_2023_8348_MOESM2_ESM.tif]

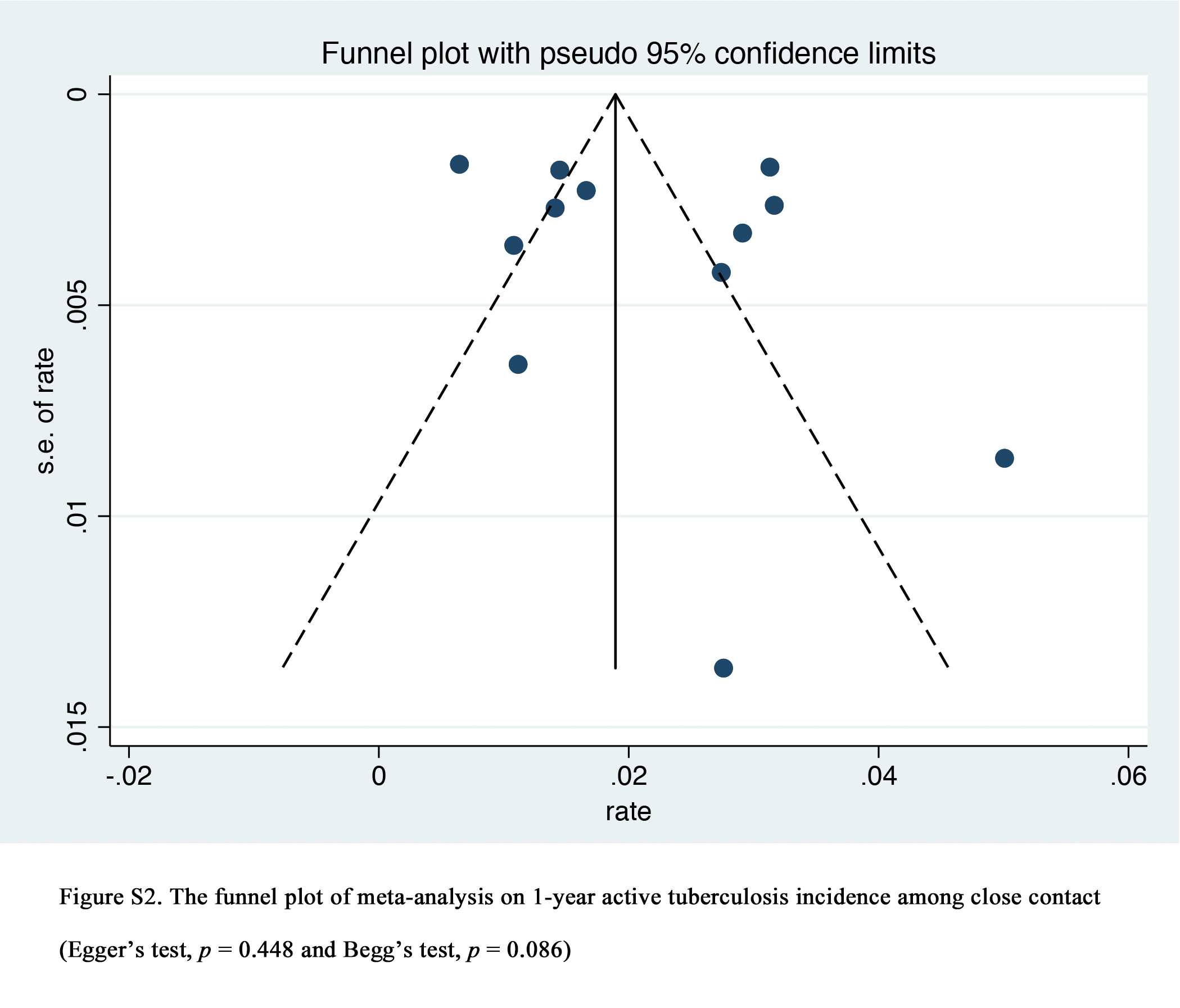

Supplement: Supplementary file 3 — Additional file 3. [file 12879_2023_8348_MOESM3_ESM.tif]
